# New Drugs, Appliances, and Things Medical

**Published:** 1891-08-01

**Authors:** 


### NEW DRUGS, APPLIANCES, AND THINGS MEDICAL.

[All preparations, appliances, novelties, etc., of which a notice is desired, should be sent for The Editor, to care of The Manager, 140, Strand, London, W.C.]

### THE "TITAN" PATENT SOAP.

Samples of this soap have been forwarded for investigation and report. The first question is, does the soap correspond to the usual tests as to goodness, freedom from objectionable excess of alkali, adulterant matters and the like? In fact, is it for the user what he, or rather we should say she, wants, that is a good washing soap? The answer is, It is. But in addition to the ordinary first-class materials the soap contains a peculiar special ingredient of a more or less volatile character. The effect of this is to get rid of dirt in a marvellous manner. The inventor has an ingenious theory as to dirt on clothes and its removal. "Every atom of dirt which adheres to clothes has a sticky envelope which fastens the dirt to them. Titan soap so acts upon this adhesiveness that it is changed in character, and the dirt atom merely rests on and not adheres to the fabric." The motion of the boiling water then carries off the dirt, which is removed by the boiling soap solution entering every crease and fold. Now let us see what this leads to. It means that no friction is required as in the old method of washing. Of the importance of this in prolonging the life of the washed materials the careful housewife will be fully aware, as there is no tearing of the clothes, and rough and fine things can be cleansed together. Again, it is particularly suited for flannels and woollens, as the oily, bland nature of the soap keeps the fibres soft and free from harshness. Of course we do not mean that these last named articles should be boiled, but washed in tepid water. Another very ingenious idea of the patentee is seen in the soap bar. It is divided by grooves into measured portions. Each part when cut up is to be dissolved in two gallons of water. There is thus an absolute guide as to quantity to be used, and thus no waste ensues, another enticement for the careful housekeeper. The inventor claims that during the process of boiling the before mentioned essential oil vapourises, and then we suppose in the presence of steam forms peroxide of hydrogen and so bleaches the clothes naturally and without the destructive aid of chemicals. The vapour is also disinfectant and deodorant, leaving the clothes refreshingly sweet. We have said we find the soap of pure manufacture, consequently coloured goods can be washed with it. Again, time and money can both be saved by its use, because there is no preliminary steeping and rubbing, and because the time required in boiling is considerably less. The clothes are simply put dry into the copper which has the soap solution ready for boiling in it. This will appeal to those who have to wash hospital and sick-room linen. There is no handling of foul clothes. After fifteen to twenty minutes' boiling the clothes are ready to be rinsed and dried, a great improvement on the old process of laboriously rubbing the clothes all over with soap before boiling. We have mentioned the claims of this soap rather more extensively than usual, as besides investigating it chemically, we induced our domestics to make a trial of it. Their verdict is unanimous that it is an enormous improvement on the ordinary soap, that it is pleasant to the hands, and does not crack them, and that it leaves the clothes bright, sweet, and clean, the labour in doing this being very much less than when the usual method of washing is employed. So chemically and practically we can speak highly of this new claimant for cleansing.

A case of sciatic pain and paralysis from over use of the sewing-machine is recorded in *Le Progrès Médical* which is hardly to be wondered at. The woman afflicted was at the time of seizure twenty-seven years old. She began to use the machine at the age of fourteen, and during the following thirteen years had worked with it, using both feet, but the right more than the left, on an average fourteen hours a-day!

